# Epiploic Appendagitis and Omental Infarction as Rare Causes of Acute Abdominal Pain in Children

**DOI:** 10.3390/pediatric13010010

**Published:** 2021-02-05

**Authors:** Federica Bianchi, Carlos Leganés Villanueva, Núria Brun Lozano, Ilaria Goruppi, Susana Boronat Guerrero

**Affiliations:** 1 Pediatric Surgery Unit, Department of Pediatrics, Hospital de la Santa Creu i Sant Pau, 08041 Barcelona, Spain; cleganes@santpau.cat (C.L.V.); nbrun@santpau.cat (N.B.L.); goruppi.ilaria@gmail.com (I.G.); 2 Pediatric Unit, Department of Pediatrics, Hospital de la Santa Creu i Sant Pau, 08041 Barcelona, Spain; sboronat@santpau.cat

**Keywords:** acute abdominal pain, appendicitis, pediatric, appendages, abdominal fat necrosis

## Abstract

Omental infarction and epiploic appendagitis are rare causes of acute abdominal pain in the pediatric population. Radiological evaluation is necessary to establish a specific diagnosis and to differentiate appendicitis from these conditions as they can be often managed conservatively without surgical intervention.

## 1. Introduction

Abdominal pain is a common complaint in children presenting to emergency department. The differential diagnosis includes serious surgical pathologies.

Omental infarction (OI) and epiploic appendagitis (EA) are included in primary abdominal fat necrosis and are rare causes of abdominal pain in children [1,2,3]; because the clinical presentation is not pathognomonic, they can mimic acute appendicitis (AA).

Early identification by imaging studies may prevent unnecessary surgical procedures [4,5].

We report two cases of primary abdominal fat necrosis, aiming to spread awareness to physicians regarding the clinical presentation and management of this unusual cause of pediatric acute abdominal pain. A review literature was performed.

## 2. Case Report 1

A previously healthy 12-year-old boy presented to our pediatric emergency department with an abrupt onset of colic pain in the right flank and inguinal fossa. There were no other associated symptoms.

On examination, he was afebrile with normal vital signs. He was well-hydrated and had neither mouth ulcers nor conjunctival icterus. His weight, height and body mass index were 60 kg, 150 cm and 26.6 (overweight range), respectively. Cardiovascular and respiratory examinations were normal. The abdomen was soft with mild and diffuse tenderness on deep palpation in the right abdominal quadrants but without rebound tenderness or guarding.

Abdominal ultrasonography (US) and the laboratory work-up were unremarkable. Therefore, the patient was elected to conservative treatment with an oral non-steroidal anti-inflammatory drug at home.

However, two days later the patient returned to the emergency department describing his abdominal pain as persisting despite medication. The laboratory work-up was unremarkable with only a mildly increased level of serum C-reactive protein (42.9 mg/L).

**Figure 1:**
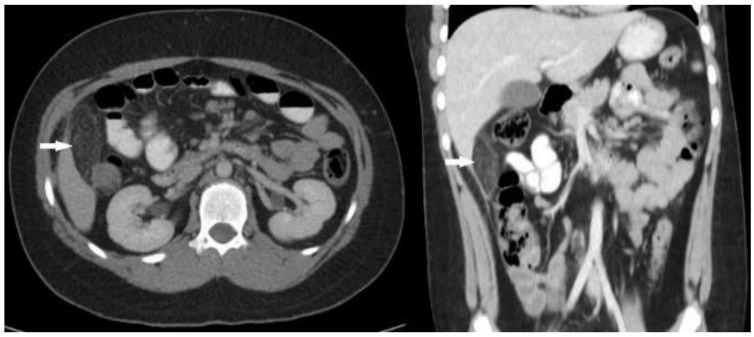
Axial (**left**) and coronal (**right**) CT images show an oval fat-attenuated mass (arrows) below the liver suggestive of OI.

A repeat US was inconclusive of acute appendicitis and a computed tomography (CT) scan was requested as a second-line diagnostic imaging tool. This revealed a hyperechoic mass below the liver with an area of approximately 6 cm (maximum diameter) of reticular infiltration of the omental fat. The CT scan well characterized the fatty nature of the lesion and was highly suggestive of OI (Figure 1).

The patient was discharged home with an oral anti-inflammatory therapy for five days and, at six months follow-up, he remains symptom free.

## 3. Case Report 2

A 16-year-old previously healthy girl presented to the pediatric emergency department with a one-day history of worsening abdominal pain. Her body mass index was normal. She described the pain as aching in character, occurring intermittently several times a day, lasting for several hours and localized to the right and left lower quadrant. No triggering or alleviating factors were identified including eating and defecation. The only associated symptom was nausea. On physical examination, she was afebrile with normal vital signs. She was well-hydrated and had neither mouth ulcers nor conjunctival icterus. Cardiovascular and respiratory examinations were normal. The abdominal examination was significant for tenderness to palpation in the lower quadrants. There were normal bowel sounds and hepatosplenomegaly was not present. The laboratory tests were normal.

**Figure 2:**
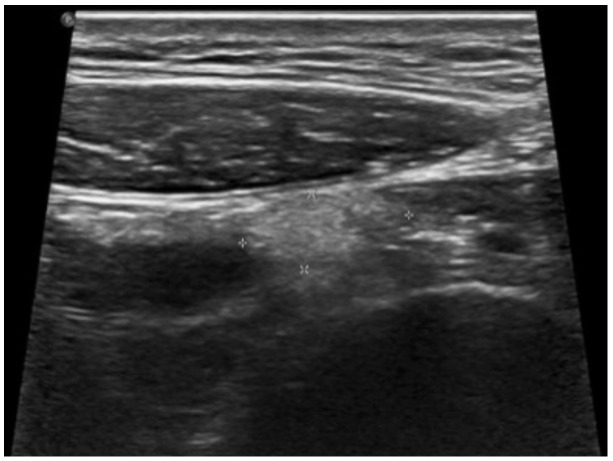
US image shows a round hyperechoic mass (crosses) behind the rectus muscle anterior to the cecum.

An abdominal US was performed and a diagnosis of EA was rendered. A fat-attenuated nodular lesion measuring 1.3 cm in diameter was observed anterior to the cecum (Figure 2). The appendix was normal and a right ovarian cyst was found.

The patient was managed conservatively with an oral anti-inflammatory therapy and she was discharged home; her symptoms resolved over 48 h with a return to a full regular diet. She was seen in follow-up two weeks after discharge from the hospital and she remained asymptomatic. She has had no recurrence of abdominal pain in the eight months since hospitalization.

## 4. Discussion

OI and EA are included in primary abdominal fat necrosis and are rare causes of abdominal pain in children.

Although the first report of OI was in 1896 by Bush [6] and the first description of EA was by Ghosh and Bilton in 1968 [7], there are not many published reports about these diseases involving children. With the advent of imaging modalities, more than half of these reports have been published in the last 25 years. Table 1 and Table 2 list the pediatric reports of primary OI and EA described in the literature from 2000 to today. The review includes 169 and 21 pediatric patients affected by OI and by EA, respectively.

Omentum is a fold of peritoneum that has a protective function for the intestinal tract in case of inflammations or injury. OI is a rare pediatric condition; it accounts for approximately 15% of all cases reported [8] and it occurs in 0.1% of children evaluated for AA. It is reportedly more common in men with a male to female ratio of 2:1 [9]. Obesity is a risk factor in the pathogenesis and the shortage of fat tissue in children explains the lower incidence in this population [10]. When twisted on itself, it can lead to infarction and necrosis of omental tissue; it occurs more often on the right side margin of the omentum. In 90% of cases involving right epiploic vessels, the arterial blood supply is more tenuous [11].

Epiploic appendages are small fat tissues located on the antimesenteric surface of the colon on the teniae. They are pedunculated and present on the entire length of the colon except the rectum where they are absent. Their size ranges from 0.5 to 5 cm with the largest ones located near the sigmoid colon and cecum. Their exact function is still unknown; probably, they have a protective function for the colon.

Like OI, EA occurs with torsion or thrombosis of arterioles feeding the appendages. It is a rare condition in the pediatric population without sex predilection [3]. In adults, it is more frequent in obese males in their fourth or fifth decade [12].

Primary abdominal fat necrosis is an uncommon cause of non-specific abdominal pain and is commonly described as acute in onset and steady in character. In epiploic appendagitis, associated manifestations are sporadic such as nausea, vomiting, anorexia and fever but they are reported in 50% of the cases of OI [13]. In our cases, only the patient with EA presented nausea.

The non-specific clinical findings cause confusion at the diagnosis. Appendicitis, acute cholecystitis and, in adult patients, diverticulitis are the most frequent misdiagnoses.

In EA, the pain is localized in the affected area; it is most frequent in the lower left quadrant (76%) followed by the right lower quadrant (20%) [14]. In fact, more than half of epiploic appendages are located in the recto-sigmoid colon. A few authors retain that pain in children is more frequently localized to the right lower quadrant and for this reason the pain mimics AA. The majority distribution of epiploic appendages in the cecum and in the sigmoid colon explains the side of pain [3]. In the OI, the pain is on the right side in 80% of cases. The clinical findings described coincide with the examination of the two cases reported.

A physical examination can mimic an acute abdomen; however, a lack of elevation in inflammatory markers or fever is suggestive of a more benign process. Neutrophilia can be detected in two-thirds of cases of OI [11].

Before the widespread use of US and CT scans, EA and OI were most commonly diagnosed at surgery. The advent of modern sophisticated imaging tools has significantly decreased the number of unnecessary surgical explorations and allowed an easier diagnosis of these conditions. However, in the last 20 years, up to 32% of cases of OI and 19% of EA are diagnosed intra-operatively, as indicated in Table 1 and Table 2.

US, CT scans and MRI can be used in the diagnosis. Both conditions present very similar imaging. OI is larger in size (3.5–7.0 cm) [15].

On US examination, which are often used to evaluate abdominal pain in children, EA is seen as a non-compressible hyperechoic mass adjacent to the colon wall with an absence of color flow on a Doppler. If the mass is within the omentum or adheres to the abdominal wall, it is a case of OI. In this case, the increased vascularity can be detected around the lesion on the color Doppler valuation due to the surrounding inflammatory reaction [2].

In our institution, US is the first imaging method used for suspected appendicitis; however, in the case of an equivocal study, a CT scan is performed.

On a CT scan, normal epiploic appendages are not seen. EA results are seen like an antimesenteric fat mass with hyperdense rim, adjacent fat stranding, a thickening of the colon wall and a specific central dot sign, which represents the central high attenuation due to the thrombotic vascular structure in the center of the epiploic appendages [16]. CT is the gold standard for the diagnosis of OI; it is seen like a well-circumscribed, inflammatory mass located between the anterior abdominal wall and the right colon [1,3].

MRI is not a well-studied imaging modality for EA or OI; however, findings are similar to a CT scan. EA is seen like a mass on the colon wall; the central portion is hyperintense on T1- and T2-weighted images, the peripheral rim is hypointense on T1- and T2-weighted images and the central dot sign appears hypointense on T2-weighted images. Absence of this sign does not eliminate the possibility of EA but the presence is a specific indicator of the pathology [17].

MRI permits the avoidance of the potential side effects of ionized radiation exposure but the low possibility of performing this exam in an emergency department and the higher cost play against the application of MRI for a differential diagnosis. A CT scan is the most widely used abdominal imaging adopted in an acute setting.

The clear disadvantages of US include that is operator-dependent, it is limited by large amounts of bowel gas and the images provided cannot always be readily interpreted by clinicians. On the other hand, the major disadvantage of a CT scan is its use of ionizing radiation.

Diagnostic imaging plays an important role in determining whether the disease should be surgically or medically treated. In our pediatric department, we promote the use of US, keeping EA and OI for the differential diagnosis of acute abdominal pain so that the US signs can be acknowledged. Only if there are doubts do we use second level exploration such as a CT scan. In the pediatric patient, US is the imaging of choice as it avoids the risk of ionizing radiation.

In our cases, the first approach was US examination, which was diagnostic in the case of EA and a combination of US and a CT scan was used in the case of OI.

EA and OI are benign, self-limiting conditions; it can be difficult to distinguish these diseases but in both situations, the management is the same. When the diagnosis is certain, they can be treated conservatively with intravenous fluids, analgesics and non-steroidal anti-inflammatory treatments. The use of antibiotics have been discussed, mainly in OI. The clinical symptoms usually resolve within two weeks and CT findings resolve after more than six months [3,18].

In the review performed, the management was conservative in 60% and 36% of EA and OI, respectively (Table 1 and Table 2). We underline the fact that the percentage of patients undergoing surgery for OI decreases from 63 to 33% if the cases undergoing surgery for suspected AA are excluded.

A few authors support early operative management in OI, described as associated with a faster recovery, better pain control and prevention of complications such as abscesses and even sepsis if a necrotic segment of omentum is left in the abdomen [8]. In our cases, the conservative management based only on oral anti-inflammatory therapy was successful. We believe that a conservative treatment is more adequate especially in children, avoiding the risk of surgery, postoperative adhesion and bowel obstruction. However, no comparative study demonstrates a significant difference in outcome following operative and conservative management [3].

Complications of EA and OI are rare and include adhesions, abscess formation, peritonitis, bowel obstruction, intussusceptions or intraperitoneal loose body. In this case, such as in the case of uncertain diagnosis or recurrence, surgical intervention is necessary; in this case, a laparoscopic approach is preferred [12,19]. No complications were onset from the literature review except for intractable pain or persistent peritoneal signs in five cases (1 EA, 4 OI). The patients initially assigned to conservative treatment subsequently required surgery.

## 5. Conclusions

Although EA and OI are usually seen in adults, they are a rare cause of acute abdominal pain in the pediatric population. They should be considered in the differential diagnosis of acute or recurrent right lower quadrant pain in children.

The diagnosis of OI and EA is challenging and in the pediatric population US is the imaging of choice as it avoids the risk of ionizing radiation. Considering these conditions, the differential diagnosis has the potential to decrease the number of CT scans because of the benign nature of the disease. CT scans should be reserved only if the US result is negative or inconclusive. Identifying a normal appendix by imaging is fundamental in this scenario.

A high index of suspicion and an early identification of primary abdominal fat necrosis appears to prevent unjustified surgical procedures in children.

## Figures and Tables

**Table 1:** Summary of reported cases of children with omental infarction.

**Authors** **(Year Reported)**	**Patients No.**	**Sex**	**Average Age** **(Range)** **in Years**	**Preoperative Diagnosis with Imaging Studies**	**Other Preoperative Diagnosis**	**Approach**	**Complications**	**Intraoperative Diagnosis**
Helmrath et al. [1], 2001	18	M (12) F (6)	7.5 (2–13)	US (4) CT (2)	AA (6)	Surgery (18)	-	+(12)
Grattan-Simth et al. [2], 2002	9	M (5) F (4)	8.0 (3–11)	US + CT (5)	Undiagnosed (4)	Surgery (8) Conservative (1)	-	+(4)
Houben et al. [3], 2003	1	M	8	-	AA (1)	Surgery (1)	-	+(1)
Nagar et al. [4], 2003	2	M (2)	9.0 (8–10)	US + CT (2)	-	Conservative (2)	-	
Varjavandi et al. [5], 2003	4	M (3) F (1)	12.5 (10–14)	CT (3)	Undiagnosed (1)	Surgery (4)	-	+(1)
Sakellaris et al. [6], 2004	2	M (2)	8.0 (7–9)	-	AA (2)	Surgery (2)	-	+(2)
Loh et al. [7], 2005	12	M (10) F (2)	9.0 (4–11)	CT (4)	AA (8)	Surgery (12)	-	+(8)
Lee et al. [8], 2005	6	M (5) F (1)	8.8 (5–11)	CT (2)	AA (3) Undiagnosed (1)	Surgery (6)	-	+(4)
Coulier [9], 2006	1	M	10	US + CT (1)	-	Conservative (1)	-	
Aoun et al. [10], 2006	1	M	11	US + CT (1)	-	Conservative (1)	-	
Van Kerkhove et al. [11], 2006	1	F	6	-	Undiagnosed (1)	Surgery (1)	-	+(1)
Fragoso et al. [12], 2006	1	M	9	US + CT (1)	-	Conservative (1)	-	
Zargar et al. [13], 2007	1	M	6	-	AA (1)	Surgery (1)	-	+(1)
Foscolo et al. [14], 2007	1	F	6	US + CT (1)	-	Surgery (1)	-	
Agresta and Bedin [15], 2007	2	M (2)	11.5 (9–14)	-	AA (2)	Surgery (2)	-	+(2)
Nubi et al. [16], 2009	10	M (6) F (4)	9.1 (5–14)	US + CT (2) CT (8)	-	Surgery (3) Conservative (7)	+(3) *	-
Rimon et al. [17], 2009	19	M (10) F (9)	9.3 (4–17)	US (5) US + CT (8) CT (1)	AA (5)	Surgery (5) Conservative (14)	-	+(5)
Yang et al. [18], 2010	7	M (6) F (1)	10.9 ± 0.6	US (1)	AA (6)	Surgery (7)	-	+(6)
Ventham et al. [19], 2010	1	M	16	-	AA (1)	Surgery (1)	-	+(1)
Bradley and Adger [20], 2010	1	M	8	CT (1)	-	Conservative (1)	-	
Gosain et al. [21], 2010	10	M (9) F (1)	8.9 (7–11)	CT (9)	AA (1)	Surgery (10)	-	+(1)
Kambouri et al. [22], 2011	1	M	10	-	AA (1)	Surgery (1)	-	+(1)
Tsunoda et al. [23], 2012	1	F	9	CT (1)	-	Conservative (1)	-	
Sandusky and Herliczek [24], 2012	1	M	11	1 (MRI)	-	Conservative (1)	-	
Wertheimer et al. [25], 2014	1	M	12	CT + MRI (1)	-	Conservative (1)		
Estevão-Costa et al. [26], 2014	8	M (4) F (4)	8.5 (6–13)	US + CT (4) CT (2)	AA (2)	Surgery (2) Conservative (6)	-	+(2)
Armas Álvarez et al. [27], 2014	3	M (2) F (1)	7.3 (6–9)	US (2) CT (1)	-	Conservative (3)	-	
Hamchou et al. [28], 2014	2	M (2)	10.5 (10–11)	-	AA (2)	Surgery (2)	-	+(2)
Koay and Mahmoud [29], 2015	1	F	7	US (1)	Simultaneous AA (1)	Surgery (1)	-	
Arigliani et al. [30], 2016	1	M	3	-	AA (1)	Surgery (1)	-	+(1)

M: male, F: female, US: ultrasonography, CT: computerized tomography, MRI: magnetic resonance imaging, AA: acute appendicitis, *: surgery for intractable pain.

**Table 2:** Summary of reported cases of children with epiploic appendagitis.

**Authors** **(Year Reported)**	**Patients No.**	**Sex**	**Average Age** **(Range) in Years**	**Preoperative Diagnosis with Imaging Studies**	**Other Preoperative Diagnosis**	**Approach**	**Complications**	**Intraoperative Diagnosis**
O’Rourke et al. [31], 2001	1	M	9	CT (1)	-	Surgery (1)	-	
Hurreiz and Madavo [32], 2005	1	F	16	-	AA (1)	Surgery (1)	-	+(1)
Gupta and Kumar [33], 2008	1	M	8	-	AA (1)	Surgery (1)	-	+(1)
Christianaki et al. [34], 2009	1	F	8	-	Intractable abdominal pain (1)	Surgery (1)	-	+(1)
Fraser et al. [35], 2009	1	M	8	CT (1)	-	Surgery (1)	-	
Matsunaga et al. [36], 2010	1	F	5	US + CT (1)	-	nr	nr	
Goh and Rudolph [37], 2011	1	M	17	CT (1)	-	Conservative (1)	-	
Rashid et al. [38], 2012	1	M	7	-	AA (1)	Surgery (1)	-	+(1)
Cho et al. [39], 2014	1	F	8	CT (1)	-	Conservative (1)	-	
Toprak et al. [40], 2014	1	F	8	CT (1)	-	Conservative (1)	+(1) *	
Redmond et al. [41], 2015	1	M	9	CT (1)	-	Conservative (1)	-	
Joshi et al. [42], 2015	3	M (2) F (1)	15.3 (15–16)	US (1) CT (1) MRI (1)	-	Conservative (3)	-	
Ullah et al. [43], 2016	1	M	10	CT (1)	-	Surgery (1)	-	
Boscarelli et al. [44], 2016	2	M (2)	6.0 (5–7)	MRI (2)	-	Surgery (1) Conservative (1)	-	
Ozturk et al. [45], 2018	3	M (2) F (1)	14.0 (10–17)	CT (3)	-	Conservative (3)	-	
Nhamoucha and Bouabdellah [46], 2018	1	M	14	US + CT (1)	-	Conservative (1)	-	

M: male, F: female, US: ultrasonography, CT: computerized tomography, MRI: magnetic resonance imaging, AA: acute appendicitis, nr: not reported, *: surgery for intractable pain.

## Data Availability

No new data were created or analyzed in this study. Data sharing is not applicable to this article.

## Abbreviations

OI	Omental infarction
EA	Epiploic appendagitis
AA	Acute appendicitis
US	Ultrasonography
CT	Computed Tomography
MRI	Magnetic resonance imaging
